# International Appraisal of Nursing Culture and Curricula: A Qualitative Study of Erasmus Students

**DOI:** 10.1155/2016/6354045

**Published:** 2016-01-28

**Authors:** Jose Siles Gonzalez, Carmen Solano Ruiz, Angela Gaban Gutierrez

**Affiliations:** Culture of Care Group, Department of Nursing, University of Alicante, 03690 Sant Vicent del Raspeig, Spain

## Abstract

*Introduction*. Globalization of knowledge has emphasized the need to promote the adoption of international exchange programs in nursing. Nevertheless, the differences in cultural, educational, and structural schemes have challenged the mutual appraisal and understanding of the nursing curricula between countries. Research on nursing curricula should allow performing an analysis of different cultural idiosyncrasies in which educational and health institutions are found. These studies would contribute valuable information to the educative and organizational systems and their cultural variability.* Objective*. To examine the experiences of nursing students on international exchange programs.* Methods*. Comparative Education was taken as theoretical background. The clinical practice diaries of seven Spanish Nursing Erasmus students (a European international exchange program) were used as field journals. These students undertook their placements in the United Kingdom. A content analysis was carried out to find major themes.* Results*. Data extracted from the students clinical practice diaries indicated cultural, educational, and structural differences between countries. Most students reflected the hidden curriculum in their diaries, writing about affective, ideological, personal, and social elements and beliefs.* Conclusions*. The students' experiences on international exchange programs were found to be sources of interest to clarify the ideological and cultural connections that underlie educational and health systems.

## 1. Introduction

Globalization of knowledge has emphasized the need to promote the adoption of international exchange programs in nursing, both in a theoretical and in a practical context [1]. Among those programs, the Erasmus student exchange program, which takes place within the European Union, has been recognized as a tool to promote culturally competent care [2], to develop self-confidence, and to enable linguistic, cultural, and social growth [3].

The establishment of the European Higher Education Area (EHEA) and the application of the Tuning Educational Structures in Europe project are bound to play a crucial role in the homogenization of the educational systems. Nevertheless, the differences in cultural, educational, and structural schemes have challenged the mutual appraisal and understanding of the nursing curricula of the different European countries [4].

Underwood [5] associated cultural diversity to lifestyles, health problems, and health systems. This concept describes a complex reality that must be taken into account to consolidate the EHEA. Furthermore, the weight of the ideological component in the way nursing curricula are settled must be considered, since the clinical settings where students develop their practice might have cultural and ideological connotations that would differ according to the country where the clinical training is conducted [4].

Hence, research on nursing curricula should allow performing an analysis of different cultural idiosyncrasies in which educational and health institutions are found. These studies would contribute valuable information to the educative and organizational systems and their cultural variability [4, 6].

Finally, teaching and learning processes should focus on students as stated in the principles of the EHEA. They should enhance practice reflection and critical thinking and promote participating in learning schemes to channel the students' voices throughout the teaching-learning process [4].

## 2. Objective

The objective of this study was to examine the experiences of nursing students on international exchange programs.

## 3. Methods

### 3.1. Comparative Education

The theoretical assumptions underlying this study were derived from Comparative Education [7] and the cultural variations that produce different ideological perspectives in nursing theory and practice.

Comparative Education is a pedagogical discipline that has always been in transition, since education systems are dynamic [8]. It has contributed to informing the characteristics of the educational systems of the European community [9] and to understanding the differences and similarities in the preregistration nursing education in Australia, New Zealand, the United Kingdom, and the United States of America [10].

Comparative Education studies in nursing have appeared recently in Spain: an initial study offered a conceptual and methodological clarification that described nursing as a transnational profession [6]. A second study employed clinical practice diaries to reach typical goals related to Comparative Education, such as overcoming the dichotomy between theory and practice [4].

These researchers employed clinical practice diaries as a tool for practical reflection, critical thinking, and analysis of students' clinical experiences from two perspectives: cultural (complexity studies in anthropology) and educational (Comparative Education) [1]. In an international framework using narrative and clinical practice diaries, the controversial matter of self-assessment and peer assessment as an essential part of the stipulations issued by the EHEA arose [4].

### 3.2. Clinical Practice Diaries

The clinical practice diary is widely used as a field journal in Educational Ethnography. It is neither an assessment* sensu stricto* nor an additional assignment for the student, since it merely reflects the student's daily practice and observations. It has the nature of a personal document that stores the formative history or the daily experience in class and serves as a tool for communication with professors [11]. Due to its nature, this tool is used to store complex information that can provide data on the level of persistence, capacity of synthesis and analysis, critical thinking, interest in the progression of the learning process, personal organization, hierarchy of ideas in making notes, the level of understanding of contents, resolution processes of the activities, and use of studying and learning procedures.

Some authors have considered the clinical practice diary as a natural narrative support [12] and have stated that biographical and ethnographic materials have enormous potential for reflection in practice [13] that promote understanding and the explanation of phenomena [14]. Clinical practice diaries have also demonstrated the relevance of reflection in practice through the act of writing and reading classroom experiences, especially in the formation of critical and reflective practitioners [15, 16].

The literature has emphasized advantages such as the evaluative functionality of the daily self-assessment, the reflection about action in the teaching and learning process, and the feedback clinical practice diaries that provide for educational practice [17]. Porlán highlighted the usefulness of this class record to enhance observation and analysis of practice [18] and Vermunt pointed out the relationship between learning patterns and personal and academic factors [19]. This issue is particularly relevant when the context changes and sets off in the student the need to adapt to a new lifestyle, as in the case of pupils on international exchange programs.

### 3.3. Sample

Convenience sampling was performed. The participants were 7 Erasmus Spanish students: three males and four females, who moved to the United Kingdom in 2013 for a ten-week placement. The mean age was 20.8 years. All of them were enrolled in their 3rd year of the Nursing Degree.

The Erasmus program in the United Kingdom took place in hospitals located in Aberdeen, Glasgow, and Newcastle, all of them belonging to the National Health Service (NHS).

### 3.4. Design, Data Collection, and Data Analysis

The technique used for data collection consisted of a clinical practice diary used as a field journal, with the students as participant observers [20]. Eleven students received training on what a clinical practice diary is and which elements should be reflected. They were also shown examples and a practical exercise was performed.

Once they returned from their stays abroad, the students were offered to enter the study. Seven of them accepted and four declined. All participants were informed about the aims of the study and confidentiality of data was guaranteed.

An initial reading of all the clinical practice diaries favored the understanding of the overall meaning of the dataset. After processing the seven clinical practice diaries through a text categorization by significant fragments, the codes were grouped into four major themes, following content analysis methods [20].

Using qualitative software ATLAS.ti 7 facilitated nonlinear reading of the clinical practice diaries. This instrument was intended to make a hypertext reading, as provided by Roland Barthes and collected by Nelson (coiner of the term “hypertext”): to be able to select linked text fragments, not line by line or paragraph by paragraph, but by units of meaning or section by section (what Barthes called* lexia* by* lexia*) [21].

## 4. Results and Discussion

After data analysis, four major themes were established: “adaptation issues,” “differences in the educational system,” “structural differences in nursing organizational schemes,” and “enriching overall experience.” The perceptions of adaptation issues and all the structural differences in nursing organizational schemes were consistent with the experiences of Spanish nurses working in the United Kingdom [22]. The experiences with the language barrier and the enriching overall experience were also consistent with the experiences of other Erasmus students [3].

Most students used the clinical practice diary as a document to reflect the hidden curriculum, writing about affective, ideological, personal, and social elements and beliefs. Some of the students used it as a tool for academic reflection, employing it as a reference book. Both cases indicate the diversity of functions and utilities of the clinical practice diary previously described: reflection on educational practice [15] without detaching it from personal and social levels [1, 19].

### 4.1. Theme  1: Adaptation Issues

The main problem students faced had a direct link with language. Most students thought that their English needed to improve and perceived language as a major barrier to communicate with the patient or with the team and to fulfill their responsibilities. The language barrier of Spanish students towards their practice abroad has been noted before [23].

*My problems with language are still there. I still do not understand people […] if you really want to benefit from the experience the required language level should be somewhat higher.*

This communication problem is not only resolved with a better understanding of the language. The communication process (including nonverbal communication) is a source of anxiety and distress for students according to their personal, social, and academic characteristics [1, 5].

Some students emphasized the kindness professionals and patients showed towards them, serving as a primary driving force for motivation and learning.

*It is striking how all the staff cares about you and worries about if you understand everything they're explaining to you.*

*At all times I felt protected, helped and motivated by all the professionals I've worked with.*

*Patients in the United Kingdom are in general much more grateful and polite, compared to Spanish patients. They appreciate and value all the time you spend with them, and they always say thank you several times before you leave the room.*

*I'm surprised by the kindness and self-confidence that many English people have, as opposed to the distorted cold view we have of them.*

On the other hand, the ignorance of the routine was perceived by some students as a source of stress that they overcame as time passed by.

*The first day I would arrive pretty nervous, especially since I knew nothing about how things worked there. But little by little I got to know the system, I started to let go and dealt with it a lot better.*

Not understanding the means and ways of working is a factor that impacts students and leads to anxiety and insecurity, especially in the beginning of their clinical practices. The process is worsened when clinical training is conducted in other countries. The fact that the students identified this problem demonstrated the functionality of the clinical practice diary in daily practice to enhance critical reflection, necessary for self-assessment [4].

### 4.2. Theme  2: Differences in the Educational System

In the United Kingdom, each student was assigned a mentor to each unit or clinical setting to supervise the student's learning, progressively evaluating him/her and pointing out strengths and weaknesses. The mentor assigned tasks and promoted adaptation to new clinical settings using guided routines. All students described their mentors as people involved in their evolution and referred rapport.

*The mentor and the university tutor maintain a continuous communication through the assessment notebook.*

Mentors typically display attributes that favour a clinical or academic focus. This affects overcoming or not the theoretical (classroom) and practical (health center service) dichotomy, an international goal of nursing curricula. This significant issue demonstrates the usefulness of Comparative Education [24], even at a noninternational level.

The assessment process was identified as similar to the one carried out at the home university, with the sole difference that “*it is a paper form*” and the assessment was done “*in the middle and the end of the internship*.” The evaluation was performed in three phases. Students and mentors met to agree to new targets, to assess which ones have been accomplished and which have not, and to promote self-evaluation and reflection on learning. The assessment done in the middle of the internship is regarded as a guide for the student.

*It is useful to show the students about whether to try harder or to congratulate them if they're meeting the expectations.*

The British curriculum is characterized by giving importance to humanistic subjects related to nursing care and less value to subjects considered fundamental in Spain, like anatomy or physiology. Internships have a longer duration and, once graduated, the newly registered nurses must carry out a compulsory adaptation period that will monitor their practice, under the supervision of more experienced staff.

Other differences found were the entrance process for the university, which is an interview and not a governmental exam; the compensation of the internship, not remunerated in Spain; and the complete funding of the career.

### 4.3. Theme  3: Structural Differences in Nursing Organizational Schemes

The foremost theme in all the clinical practice diaries was the minor technical training of the lowest bands of nurses in the United Kingdom. It is quite recurrent in the narratives the fact that they do not perform invasive and some noninvasive procedures unless they have received additional training.

*One of the things that had drawn the attention of my tutor is that we do not have to pass a course to learn how to take blood, but we learn it during our internship, since they do have to do it.*

On the other hand, the high nursing specialization and the existence of bands and different levels of nursing surprised the students. Nurses are grouped into three broad categories: general staff nurses, nurse practitioners, and sisters, the range of responsibilities being the main difference among them. For example, the nurse practitioner may prescribe medication, and the sister handles the supervision of the unit.

The students described practitioner nurses as much more autonomous in their work, as substantially updated on procedures, and with a high capacity of care management. Plus, this group organized expert committees.

*There are nurses specialized in ulcers that form a committee, responsible for assessing and guiding the dressing treatment of those patients with an ulcer.*

The students also believed that caring was more geared towards patient comfort and more individualized, something that was observed in the helpful attitude of the team and the ongoing communication with the patients to cover their needs.

*To be at their service in all their needs, either bringing towels, food that has been left in the fridge, helping them changing their position, serving more coffees and teas, meals, etc.*

The students highlighted the importance of a comprehensive and detailed record, where all the information related to the patient and nursing activities was registered through standard forms, charts, scales, or score sheets.

*It becomes quite stressful to have so many scales for everything, but in the end when you get used to it you see it's really necessary.*

The students believed that care planning was better, probably due to the completeness in the register. They indicated that there were care plans for each problem or procedure and that care is delivered in a very homogeneous way and quite adjusted to the theory. They highlighted the continuous assessment above all other stages of the nursing process. They also referred to the public inspections of hospitals and audit of care plans that the National Health Service executes.

*Constantly, all units are visited by inspectors from the hospital or the National Health Service to ensure that work is done correctly. They usually ask for care plans and interview patients to assess nursing care. I think that motivates nurses to do the best job possible.*

*In the United Kingdom they may be extremists, and they want to have everything under control, minutely calculated, signed and dated.*

The latter statement is related to another phenomenon: the “double check,” associated with rigid protocols. The double check involves checking and registration by two nurses of any activity that can entail the minimal risk, as the administration of medication which remains locked, intravenous therapy, even if it is just a saline with ions or insulin, and counting drugs, particularly opioids.

Another difference at the structural level is the rigorous performing of infection control. A weekly general cleaning is done in all units, and once a month the committee in charge performs an inspection. Several of the students refer to the use of disposable plastic aprons distinguished by a colour code indicating which one should be used for each situation: yellow if there is risk of infection or in case of isolation, green to serve and collect food, blue for grooming, and white for wound dressings.

A strong focus is placed on both nosocomial disease control and quality of care. This fact can be related to the ideological component of the British health system, since, as Habermas notes, control techniques in institutions act as an audit of the technical quality of the tasks entrusted to these institutions [25]. Otherwise, the existence of strict control mechanisms in some health systems, while others just reflect it on paper with a pure appearance almost exclusively formalistic and bureaucratic, causes disorientation and anxiety in students and professionals from different health systems. This complex phenomenon can only be clarified and distinguished by applying the methods of Comparative Education [8] and critical thinking [25].

### 4.4. Theme  4: Enriching Overall Experience

The experience of the exchange program was described as hard and productive. Most of the students explained how the contrasts in nursing between Spain and Britain have made them think of the strengths and weaknesses of each system and gain a broader view of Nursing Science.

*Knowing how other health systems are has allowed me to see clearly what are the strengths and weaknesses of our own and to recognize how I can improve as a nurse.*

Several participants pointed out the personal and professional growth, especially in terms of communication with the patient, as the most significant result of their experience abroad.

*I would have never thought that three months away from home could change both the approach that I have about myself, my future as a nurse, my place, my life project and the perception that I have on many things.*

### 4.5. Implications for Practice

Reflection process and critical thinking have been explained and analyzed following the students' experiences written in their clinical practice diaries, therefore demonstrating the relevance of the clinical practice diary as a facilitator of reflection in action, peer, and self-assessment. The relationship between learning patterns and personal and academic factors, a matter of great importance when the context is different, has also been reflected in the clinical practice diary.

The combination of Comparative Education and the clinical practice diaries has shown itself as a relevant tool to clarify the ideological and cultural connections that underlie educational and health systems. It has also provided an overview for the establishment of international comparisons that will contribute to a better command of the nursing culture and curricula.

### 4.6. Limitations and Future Research

Data could have been collected up to theoretical saturation, but this was not possible because of the small sample available. Theoretical sampling could be performed to gain a deeper understanding of the analyzed cases and thus facilitate the development of and analytic frame.

Channeling the students' experiences throughout the teaching-learning process has provided a new insight into nursing culture and curricula. The comparison of narrative materials of Erasmus and non-Erasmus students, or the comparison of British and Spanish student's experiences while on exchange programs in Spain and the United Kingdom, respectively, may also enrich the understanding of the cultural values and behaviours that lie beneath the educational and health systems.

A comparison of clinical practice diaries written by students, lecturers, and other professionals (graduated nurses, social workers, psychologists, etc.) from different educational and health systems would also allow a comparative analysis in which Comparative Education methods may be useful.

Considering policy, students have emphasized the need for nursing in the United Kingdom to be less bureaucratic, while in Spain a less technical and more humanistic and patient-centered focus should be approached. These reflections should be contemplated to improve quality of care in both countries.

## 5. Conclusions

Following the Erasmus students' experiences, this study has identified some differences and similarities in the nursing educational and organizational systems between Spain and the United Kingdom and the differences and similarities in the clinical learning process of nursing students from Spain developing their training abroad.

Regarding educational features, the students highlighted the following factors, critical in the British system: the figure of the mentor and the continuous communication and good rapport, a more humanistic curriculum, and more facilities to enter university. When considering structural matters, the students found an increased variability in the hierarchy of British nurses, less technical but more humanistic care, rigid protocols and register, and, generally, a better communication and planning of nursing care.

The students described their emerging needs during their clinical training abroad. Particularly significant is the communication problem, which does not begin or end with language proficiency. The students also explained the different strategies used to cope with problems; in adopting these tactics critical thinking, reflection in clinical action, and the feeling of autonomy and participation experienced by students have been fundamental.

## References

[1] Siles González J., Solano Ruiz M. D. C. (2009). *Antropología Educativa de Los Cuidados: Una Etnografía del Aula y las Prácticas Clínicas*.

[2] Milne A., Cowie J. (2013). Promoting culturally competent care: the Erasmus exchange programme. *Nursing Standard*.

[3] Yücelsin-Tas Y. T. (2013). Problems encountered by students who went abroad as part of the Erasmus programme and suggestions for solutions. *Journal of Instructional Psychology*.

[4] Siles González J., Solano Ruiz M. C. (2012). The convergence process in European Higher Education and its historical cultural impact on Spanish clinical nursing training. *Nurse Education Today*.

[5] Underwood S. M. (2006). Culture, diversity, and health: responding to the queries of inquisitive minds. *Journal of Nursing Education*.

[6] Siles González J., Pérez Cañaveras R. M., Fernández Sánchez P. (1992). La Enfermería comparada: un instrumento para canalizar y sistematizar las experiencias y conocimientos de una profesión transnacional. *Enfermería Científica*.

[7] García Garrido J. L. (1991). *Fundamentos de Educación Comparada*.

[8] Epstein E. H. (1986). Currents left and right: ideology in comparative education. *Comparative Education Review*.

[9] García Garrido J. L. (1994). *Reformas Educativas en Europa*.

[10] Lusk B., Russell R. L., Rodgers J., Wilson-Barnett J. (2001). Preregistration nursing education in Australia, New Zealand, the United Kingdom, and the United States of America. *Journal of Nursing Education*.

[11] Woods P. (1998). *Investigar el Arte de la Enseñanza. El Uso de la Etnografía en la Educación*.

[12] de los Á Martínez Ruiz M., Sauleda i Parés N. (2002). *Las Narrativas de los Profesores, una Perspectiva Situada*.

[13] Pujadas Muñoz J. J. (2002). *El Método Biográfico: El Uso de las Historias de vida en Ciencias Sociales*.

[14] Plummer K. (1989). *Los Documentos Personales. Introducción a los Problemas y la Bibliografía del Método Humanista*.

[15] Schön D. A. (1998). *El Profesional Reflexivo: Cómo Piensan los Profesionales Cuando Actúan*.

[16] Perrenoud P. (2004). *Desarrollar la Práctica Reflexiva en el Oficio de Enseñar: Profesionalización y Razón Pedagógica*.

[17] Kemmis S. (1999). Action research and policy thinking. *Desarrollo Profesional del Docente: Política, Investigación y Práctica*.

[18] Porlán R. (2008). *El Diario de Clase y el Análisis de la Práctica*.

[19] Vermunt J. D. (2005). Relations between student learning patterns and personal and contextual factors and academic performance. *Higher Education*.

[20] Taylor S. J., Bogdan R. (1986). *Introduction to Qualitative Research Methods. The Search for Meanings*.

[21] Barthes R. (1997). *La Aventura Semiológica*.

[22] Darriba P. (2010). *Cuando la Enfermera Es la Emigrante: Experiencias de Profesionales de la Enfermería Españoles en el Reino Unido*.

[23] Goodman B., Jones R., Sanchón Macias M. (2008). An exploratory survey of Spanish and English nursing students' views on studying or working abroad. *Nurse Education Today*.

[24] Saarikoski M., Helena, Warne T. (2002). Clinical learning environment and supervision: testing a research instrument in an international comparative study. *Nurse Education Today*.

[25] Habermas J. (2010). *Ciencia y Técnica Como Ideología*.

